# The worse we feel, the more intensively we need to stick together: a qualitative study of couples’ emotional co-regulation of the challenge of multimorbidity

**DOI:** 10.3389/fpsyg.2023.1213927

**Published:** 2023-08-11

**Authors:** Andrea B. Horn, Lukas Zimmerli, Andreas Maercker, Barbara M. Holzer

**Affiliations:** ^1^CoupleSense: Health and Interpersonal Emotion Regulation Lab, University Research Priority Program “Dynamics of Healthy Aging,” University of Zurich, Zurich, Switzerland; ^2^Center of Gerontology, Healthy Longevity Center, University of Zurich, Zurich, Switzerland; ^3^Gerontopsychology and Gerontology, Department of Psychology, University of Zurich, Zurich, Switzerland; ^4^Department of Internal Medicine, Cantonal Hospital Olten (KSO), Olten, Switzerland; ^5^Psychopathology and Clinical Intervention, Department of Psychology, University of Zurich, Zurich, Switzerland; ^6^Department of Internal Medicine, University Hospital Zurich, Zurich, Switzerland

**Keywords:** couples, interpersonal emotion regulation, multimorbidity, dyadic, coping with disease

## Abstract

**Introduction:**

Being faced with multimorbidity (i.e., being diagnosed with at least two chronic conditions), is not only demanding in terms of following complicated medical regimes and changing health behaviors. The changes and threats involved also provoke emotional responses in the patients but also in their romantic partners. This study aims at exploring the ways of emotional co-regulation that couples facing multimorbidity express when interviewed together.

**Method:**

*N* = 15 opposite sex couples with one multimorbid patient after an acute health crisis that led to hospitalization were asked in a semi-structured interview about how they found ways to deal with the health situation, what they would recommend to other couples in a similar situation, and how they regulated their emotional responses. Interviews were analyzed qualitatively following open, axial, and selective coding, as in the grounded theory framework.

**Results:**

Emerging categories from the romantic partners’ and the patients’ utterances revealed three main categories: First, *overlapping cognitive appraisals about the situation* (from fighting spirit to fatalism) *and we-ness* (construing the couple self as a unit) emerged as higher order factor from the utterances. Second, *relationship-related strategies* including strategies aimed at maintaining high relationship quality in spite of the asymmetric situation like strengthening the common ground and balancing autonomy and equity in the couple were often mentioned. Third, some couples mentioned how they benefit from *individual strategies* that involve fostering individual resources of the partners outside the couple relationship (such as cultivating relationships with grandchildren or going outdoors to nature).

**Discussion:**

Results underline the importance of a dyadic perspective not only on coping with disease but also on regulating the emotional responses to this shared challenging situation. The utterances of the couples were in line with earlier conceptualizations of interpersonal emotion regulation and dyadic perspectives on we-disease. They broaden the view by integrating the interplay between individual and interpersonal regulation strategies and underline the importance of balancing individual and relational resources when supporting couples faced with chronic diseases.

## 1. Introduction

Coping with chronic diseases is a challenging endeavor. Not only many behavioral adjustments but also emotional adjustments are necessary, given the dramatic changes in life perspectives, self-image, and unpleasant experiences like pain and disability that need to be integrated (Schulze et al., 2014). This is particularly the case in patients with multimorbidity (Rijken et al., 2020). *Multimorbidity* is commonly defined as two or more chronic medical conditions co-existing in a person at the same time (Boyd and Fortin, 2010). The prevalence of multimorbidity is higher with increasing age and female gender and with low socioeconomic status (Marengoni et al., 2011; Barnett et al., 2012; Violan et al., 2014).

A large proportion of the global population, especially persons aged 65 years or older, is affected by multimorbidity (Nguyen et al., 2019). People in high-income countries are more affected than people in middle- or low-income countries, with an estimated overall prevalence of 44.3%, compared to 36.8% in low-income or middle-income countries, although heterogeneity is high (Ho et al., 2022). There are many reasons for this, such as an older population, a lower proportion of infectious diseases and higher life expectancy in populations of high-income countries.

Multimorbidity is the most common chronic condition in old age, with a prevalence in the general population of 65% among those aged 65–84 years and 82% among those older than that (Barnett et al., 2012). At Swiss university hospitals, the prevalence of multimorbid inpatients at departments of internal medicine is about 80% (Aubert et al., 2019).

Psychosocial adjustment is particularly challenging in multimorbid situations, as the regimens of multiple conditions need to be followed, demanding a high load of behavior change in the patients (Schulze et al., 2014). Furthermore, personal and medical control of the illness is often perceived as impaired, as the complexity of the health situation demands a lot not only from the patient and the persons close to them but also from the health professionals (Skou et al., 2022). A highly personalized, proactive care regimen is advocated for appropriate treatment of people suffering from multimorbidity. Accordingly, a recent study found that illness perceptions involving low perceived personal control, high levels of emotional concern, and low levels of perceived social support were indicative of the least adjusted group of high-need patients (Rijken et al., 2020). The complexity of the multimorbid situation combined with the chronic timeline makes it a particular demanding situation requiring significant psychological adjustment (Schulze et al., 2014; Rijken et al., 2020).

Recently more and more attention has been drawn to the fact that health conditions challenge not only the person with diagnoses but also their social network. In most cases the closest person in adulthood is the romantic partner. Consequently, more and more emphasis has been given in the literature on an interpersonal perspective of coping with and adjusting to disease (Manne and Zautra, 1990; Berg and Upchurch, 2007; Helgeson et al., 2018).

### 1.1. The importance of emotions and their (co-)regulation in the context of disease

Most frameworks and studies focus on adjustment to a disease as an outcome of successful coping processes, with a focus on the demands on patients entailed in management of the disease. However, adjustment is not only the result of successful coping with demands but also the result of successfully maintaining affective well-being (Schulze et al., 2014). For better functioning and for maintaining proper health behaviors, it is crucial to deal with the emotional impact of chronic diseases. This concerns patient and partner, as both are faced with a situation that is prone to trigger emotional responses.

For a long time, when investigating and intervening in adjustment processes in the context of coping with disease and in general in stressful situations, researchers have often applied a “lone man against the elements” perspective (Rimé, 2009, p. 60), implying that emotion regulation involves social processes only in childhood and possibly in older age. However, recent developments in the field of emotion regulation research support the notion that also in adulthood, co-regulation of emotions with a partner plays a prominent role in psychosocial adjustment and consequently in physical and mental health (Rimé, 2007; Butler and Randall, 2012; Bodenmann and Randall, 2013; Maercker and Horn, 2013; Coan and Sbarra, 2015).

Generally, emotions have been defined as “short-lived psychological-physiological phenomena that represent efficient modes of adaption to changing environmental demands” (Levenson, 1994, p. 124). Accordingly, multimorbidity should involve the occurrence of emotional responses in both patients and their partners, as the diseases pose a huge shift in environmental demands in the couples’ daily life and the chronicity of the situation implies that the situation is going to remain forever. Emotion regulation has been defined as the way that people deal with their emotional responses, by maintaining, decreasing, or increasing the occurrence and intensity of their emotions (Gross, 2002). Interpersonal emotion regulation or co-regulation of emotions has been defined as these emotion regulatory processes taking place in *social interaction* regulating either one or both partners’ emotional responses (Horn and Maercker, 2016; Horn et al., 2021; Dworakowski et al., 2022a,b). Gross and Thompson (2007) suggested that coping can be conceptualized as one part of emotion regulation. Emotion regulation has been seen as a broader concept, as it also includes the cultivation of positive emotions, well-being, and is not restricted to dealing with situations that challenge the individual’s resources but also applies to building resources and flourishing. There is a consensus in emotion research that more demands in the environment (i.e., stress), are commonly associated with emotional responses that are functional in the short term (Levenson, 1994). Prolonged and less adaptive emotional responses, however, are the result of maladaptive ways of emotion regulation; this can be seen as a circular process that can take place at every step of the emotion generation process (Gross, 2015). That implies that there are preventive antecedent emotion regulation strategies, such as re-appraisal, that prevent the evolving of the full-blown emotional experience. *Re-appraisal* is defined as taking a cognitive perspective on the situation, which implies a less or different emotional response. This explains the fundamental role of emotion regulation for successful psychosocial adjustment to chronic diseases: Whether patients as well as their loved ones manage to adjust to the situation is to a huge extent dependent on how successfully they deal with the functional activation of their emotional system. This is in line with current views on resilience, which also underline the importance of early functional emotion regulation strategies like reappraisal (Riepenhausen et al., 2022; Troy et al., 2023).

As emotional co-regulation implies social interaction, relational processes need to be conceptualized for a better understanding of what interpersonal processes take place during this way of emotional co-regulation. In this context, a socio-affective pathway of emotion regulation has been suggested, representing a genuinely interpersonal process that includes the establishment of psychological intimacy leading to a more positive affective situation (Debrot et al., 2013; Horn et al., 2018). Other frameworks use the terms *social proximity* (Coan and Sbarra, 2015) or *secure attachment* (Mikulincer and Shaver, 2019).

Psychological intimacy is constitutive of a shared notion of closeness in a relationship; romantic relationships are the closest relationships in adulthood (Reis and Shaver, 1988). The interpersonal process model of psychological intimacy postulates the following process for the establishment of a shared notion of closeness (Reis and Shaver, 1988): The starting point is the disclosure of personal content, which, if it is followed by a responsive reaction by the listening partner, results in the experience of being understood, validated, and cared for. This in turn constitutes a shared notion of psychological closeness. This process needs to be constantly updated in a romantic relationship, which is particularly challenging in the demanding situation of having a partner with a chronic disease or even multimorbidity. People with multimorbidity experience an exacerbation of one of the chronic conditions on a regular basis as well as acute additional illness; this adds to the complex health situation and worsens health or even threatens the life of this person. Manne et al. (2004) applied this model to the context of couples coping with disease and found that couples that manage to maintain this process and thus maintain closeness in difficult situations can benefit from that closeness by engaging in more adaptive coping and regulation strategies (Manne et al., 2004). Also, in the context of multimorbidity, sharing personal content despite the challenging situation has been shown to be important (Horn et al., 2019). Besides psychological closeness or intimacy, physical proximity and affectionate touch has been shown to improve adjustment to demanding situations (Jakubiak and Feeney, 2017). In a study assessing daily affective well-being, hugs and other ways of showing physical affection were not only associated with more positive mood in daily life; this association was also partly mediated by increases in psychological intimacy, which underlines the interrelatedness between psychological and physical closeness in romantic relationships (Debrot et al., 2013). To sum up, there is convincing evidence pointing to the importance of social relationships for successfully regulating emotions and adjusting to stressful situations. This corresponds with the widely acknowledged impact of the quality of social relationships on mortality and health (Holt-Lunstad et al., 2010, 2015).

### 1.2. Interpersonal perspectives on adjusting to disease

In the literature more and more attention has been paid to the importance of interpersonal resources in close relationships, like couples, when it comes to coping with diseases. Berg and Upchurch (2007) applied a dyadic view and described adjustment as a result of dyadic appraisals of the situation and dyadic coping with the demands the situation poses; they pointed to the importance of temporal and contextual aspects. Manne and Zautra (1990) pointed to the importance of disclosure (i.e., opening up on personal thoughts and feelings), for the establishment and maintenance of psychological intimacy when the caregiving partner provides support to the partner with a diagnosis. This view is informed by the above-mentioned concepts in relationship science of the assumed interactive processes that are involved in the establishment and maintenance of psychological intimacy (Reis and Shaver, 1988). Similarly, the Relational Cultural Coping Model (Kayser et al., 2007) frames relational processes as mutuality, authenticity, or relationship awareness as the hub of dyadic coping with disease. Further, shared appraisal of the situation and the disease, coping strategies, opportunities for growth and meaning finding are integrated in this model, which also acknowledges the importance of emotional responses in both partners.

#### 1.2.1. We-Ness

A further elaboration of the dyadic perspective is the systemic view, which zooms out from an individual perspective to the dyad as a unit. Studies informed by a systemic view on adjustment to disease support the assumed interdependency within the couple as a systemic unit: Resources like self-efficacy can transmit from the healthy partner to the patient (Rohrbaugh et al., 2004), and high levels of distress as reported by the partner (Rohrbaugh et al., 2009) seem to have a detrimental impact on health outcomes. The systemic view suggests that a person’s own resources are expanded by including the partner’s resources to a new level that is more than the sum of the individuals’ resources (Aron et al., 2004). This has also been referred to as *we-ness*, which reflects a communal orientation in the coping process (Rohrbaugh et al., 2008; Leuchtmann and Bodenmann, 2017) and the co-conceptualization of the disease as *we-disease* (Kayser et al., 2007). In the context of coping with disease, framing the disease as a shared and manageable challenges was as an example associated with better outcome in heart disease in an earlier study (Rohrbaugh et al., 2008; Rentscher, 2019). Berg and Upchurch’s (2007) framework also underlined the fundamental role of shared appraisals of the situation. Accordingly, sharing illness perceptions in couples was found to be beneficial for couples coping with disease, when for example, the amount of personal control over the disease was congruent (Sterba et al., 2008).

#### 1.2.2. Maintaining and cultivating the relationship

Besides the systemic perspective, which frames the relationship or the dyad as a unit, other views point more to the asymmetry in couples coping with disease. In the social support literature, receiving social support has been identified as a possible threat to autonomy and self-esteem; studies by Bolger et al. (2000) found that in daily life, only invisible support—support that is not perceived as such—showed short-term positive effects on affective well-being (Gleason et al., 2008). According to social equity theory, the perceived balance of receiving and providing is crucial for the maintenance of close relationships (Gleason et al., 2003). In other words, the patient’s increasing needs and the resulting impact on the couples’ daily life and emotional adjustment to the threats related to the disease lead to a well-documented and theoretically plausible asymmetry in the couple that has the potential to challenge the quality of their relationship (Kuijer et al., 2004; Manne and Badr, 2008). Here, maintenance of the perception of closeness, psychological intimacy, and a sense of equity or balance is particularly demanding for the couple. Summing up, there are threats to the relationship quality during the demanding situation of coping with disease as a couple, which in turn puts the benefits of adaptive co-regulation at risk. This requires closeness in the couple and a positive relationship quality, which in turn is associated with better outcomes in both, the patients and their romantic partners (Kayser et al., 2007; Kayser and Acquati, 2019).

#### 1.2.3. Interplay between individual and interpersonal strategies

Overall, when it comes to dealing with difficult situations, the importance of social relationships as a resource has received convincing empirical and theoretical support. However, just as in individual emotion regulation, there are more or less adaptive ways regulating affective responses together with others. Interpersonal co-regulation can also be maladaptive. There are still a lot of unanswered questions in this field, but there is support for the assumption that there is an interplay between individual strategies and resilience and the benefit of interpersonal strategies. For example, recent studies have found that when adjusting to major stressors, highly repetitive, abstract, superficial reference to negative content in dialog with the romantic partner represents an interpersonal form of the maladaptive emotion regulation strategy called *rumination* (Aldao et al., 2010) and *ruminative co-brooding* (Horn and Maercker, 2016). Other maladaptive interpersonal emotion regulation strategies are malign or negative humor (like sarcasm) or suppressing any emotional expression (Dworakowski et al., 2022a,b). These strategies serve the purpose of regulating one’s own emotional responses but do have a significant influence on relational processes—again underlying how individual regulation attempts are in interplay with processes characterizing relationship quality. In a study investigating a sample of over 1,000 participants in four countries, maladaptive interpersonal regulation was associated with worse psychosocial adjustment to the second wave of the COVID-19 pandemic, above and beyond established individual emotion regulation strategies (Dworakowski et al., 2022a,b). This could be interpreted as follows: As soon as attempts to regulate one’s own emotional responses involve dysfunctional attempts in the social domain, adjustment processes are even more impaired. In another study, fostering emotion regulation by applying a self-applied intervention to foster one’s own emotion regulation (expressive writing) resulted in more beneficial disclosure processes when sharing concerns and challenges with the partner (Horn et al., 2021). In other words, for adaptive and beneficial support and co-regulation processes, an adaptive way of dealing with one’s own emotional responses is fundamental.

To sum up, social processes are highly relevant when it comes to regulating emotions in the context of a demanding situation. These interpersonal co-regulation processes can be beneficial, as in the best case they expand the individual’s resources and fulfill basic needs for belonging and safety. However, when these social regulation strategies are dysfunctional, the result can be a double loss situation of worse individual and social adjustment.

### 1.3. The goal of this study

This study aims to investigate how romantic partners express their emotion regulatory coping efforts in their own words when it comes to their adjustment to chronic disease and multimorbidity. The goal is to create a model of couples’ individual perceptions of this emotional co-regulation process in order to heuristically inform and expand existing theoretical models of couple adjustment to disease. Following a systemic perspective of we-disease, we are interested to find what co-constructed content emerge when couples are interviewed together.

Existing quantitative research findings assessing theory-derived constructs were based on the use of self-reports on predefined constructs. With this study, we would like to further complete the picture and foster theorizing by investigating individual processes and including both romantic partners and how they refer to their affective co-regulation when talking about their own journey through the disease. For that purpose, we conducted semi-structured interviews with couples facing an acute health crisis within a multimorbid situation and asked about their individual experiences regarding their communal way of regulating emotions and adjusting to the situation.

With this we aim to deepen the understanding of communal interpersonal regulation of emotional responses to a health situation by applying a qualitative approach that will shed light on the individualized trajectories of the co-regulation of couples facing an acute crisis in the context of multimorbidity. The overarching goal here is to contribute to the development of theories in the context of behavioral markers of communal coping and to better understand categories, opportunities, and challenges when intervening with couples in a complex health situation.

## 2. Methods

### 2.1. Sample

Participants were inpatients at the Department of Internal Medicine of University Hospital Zurich and their different-sex partners. We screened inpatients between July 2013 and April 2017 for the following inclusion criteria: currently an inpatient, minimum 18 years of age, two or more chronic conditions (multimorbidity), having a romantic partner, and both patient and partner having a very good command of the German language. The goal of this research project was to investigate the dyadic impact of multimorbidity. The shared psychological feature of multimorbidity is the complexity of coping with at least two chronic conditions. The chronic and complex characteristic of this situation and its implication were to be investigated within a transdiagnostic perspective.

Both partners signed an informed consent. Exclusion criteria were dementia, addiction, pregnancy, palliative situations, or participation in another research study one month before participation in our study. The later point was in line with current research policy at the university hospital.

Of the couples fulfilling the inclusion criteria (*N* = 515), fifteen couples (male patients, *n* = 10; female patients, *n* = 5; patients aged 47 to 78, partners 53 to 80 years; mean age of patients 66.2 years (*SD* = 8.8); partners 65.5 years (*SD* = 8.6), see Table 1) agreed to take part in the study. For more detailed information of this process please refer to Horn et al. (2019). After both had signed the informed consent, an appointment was made for the interview. Prior to the interview, each patient and partner completed a baseline survey with standardized questionnaires that included sociodemographic variables and health-related questionnaires.

**Table 1:** Sample: patients’ characteristics.

	Patients		Partners	
	%	*N* or range	%	*N* or range
Age in years, mean (*SD*)	66.2 (8.8)	47–78	65.5 (8.6)	53–80
Gender				
Female	33.3	5	66.7	10
Male	66.7	10	33.3	5
Professional status				
Employed	26.7	4	40	6
Retired	60.0	9	53.3	8
Disability pension	13.3	2		
Not answered			6.7	1
Education				
Basic vocational training	13.4	2	46.7	7
Middle/higher education	33.3	5	33.3	5
College/university degree	53.3	8	20.0	3
Relationship in years, mean (*SD*)	30.9 (15.7)	9–53		
Children				
Yes	73.3	11		
No	26.7	4		
Number of chronic conditions, mean (*SD*)	5.6 (2.1)	2–10	-	-
Number of medications, mean (*SD*)	6.1 (3.6)	1–15	-	-
Reason for hospital admission				
Unspecified, fever, pain, etc.		5	-	-
Cardiovascular		2	-	-
Respiratory		3	-	-
Blood, blood forming organs, immune mechanism		2	-	-
Musculoskeletal		2	-	-
Other		1	-	-
Self-rated health	3.5 (0.8)	2–5		
Mental Health: Depressive Symptoms PHQ9	8.9 (6.28)	2–5	4.5 (4.66)	
Over cut-off 9 of medium level subclinical depressive symptoms	5	33.3	1	6.7
Self Care Index	38.43 (2.4)	31–40		

SD, standard deviation; Information on chronic conditions: 2 or more chronic conditions (= multimorbidity), number of medications per day (daily intake of drugs), reason for hospital admission, Self-rated health: Range excellent (=1) to poor (=5). PHQ9: Patient Health Questionnaire 9: Depression Screening, values range: 0–27. Self Care Index: Taken from nursing documentation at discharge. Index scoring (sum points) ranges from 10 points (fully dependency) to 40 points (full independency).

Participants reported of having a romantic relationship with their partners on the average for 30.9 years (*SD* = 15.7); 73% had children. More than half of the patients (53%) had a college/university degree; 33% had a middle or higher education. About 60% were retired at the time of the interview. Table 1 reports information on both partners.

With regard to multimorbidity, patients had 5.6 chronic conditions (mean, *SD* = 2.1) and had a mean medication intake of 6.1 drugs daily (*SD* = 3.6). The medical symptoms and diagnoses that had led to hospital admission were diverse and in a third of the cases related to unspecified symptoms such as pain, fever, or nausea. By the time of the interview, patients rated their self-perceived health status at 3.5 (*SD* = 0.8; scale from 1 = excellent to 5 = bad). On average, patients had a Self Care Index of 38.8 (*SD* = 2.4), expressing a high degree of nursing independency at the time of their discharge (see descriptions of measures below). The study was conducted in accordance with good clinical practice (GCP) and the Declaration of Helsinki. The study protocol was approved by the Cantonal Ethics Committee Zurich (PB 2018-00326).

### 2.2. Measures

#### 2.2.1. Experiences of couples faced with multimorbidity

The analyses were based on utterances made during semi-structure interviews conducted with the multimorbid patients together with their romantic partners in a bigger mixed-methods study. Clinicians of the research team and his co-workers screened for eligible patient in the electronic medical records of the hospital. Patients fulfilling the inclusion criteria were invited together with their partners to participate in the study, to fill in paper questionnaires separately and/or answering questions in a semi-structured interview together. Participants in this qualitative study represent a subsample of a larger mixed-method project in which further questionnaires were assessed that are not in the scope of this study (Horn et al., 2019).

Eligible patients and their partners were approached two to five days after hospital admission following an acute health crisis of the patient that had led to hospitalization. Most interviews took place around one to two weeks after the acute crisis, when the medical condition had been stabilized. The interviews were conducted at the Department of Internal Medicine at University Hospital Zurich, or alternatively at the patients’ home, and lasted between 9 and 32 min, with a mean of *M* = 17.4 (*SD =* 5.6) minutes.

The questions in the semi-structured interview guide (see Supplementary material) were designed to capture the experiences, perceptions, and views of the couple coping with the complex health situation for a long period of time. The interview included open questions on how the patients and their partners found ways to cope with the health situation, what they would recommend to other couples in a similar situation, what they were proud of regarding how they mastered the situation together, and how they regulated their emotional responses. The setting was designed to foster the expression of experiences of the situation as a couple and thus conducted with both partners in a safe environment.

Interviews were conducted with both partners together by psychology students at the Master’s level not suffering from chronic diseases, who were instructed to lead the conversation in a way that allowed both partners to have comparable speaking time. For example, if one partner answered the question asked and finished the sequence, the interviewer would then target the other partner and ask his or her views on it. The young master students were trained to be empathic and encouraging in their way to speak with the couples and to listening well and attentively. The students were involved in the recruiting process and had regular contact with the treating medical staff.

Prior to the interview, couples completed a baseline survey with standardized questionnaires that included health-related questionnaires that are not in the scope of this study but other quantitative research questions. Results of that questionnaire data were published earlier (Horn et al., 2019). Prior to data collection we conducted a pilot study for testing the procedures and ensuring that the interview setting fostered expression of couple experiences. To ensure data protection, all participants’ data were pseudonymized. The recorded and pseudonymized interviews were transcribed verbatim. Analyses were conducted relying on coding in text and table formatted documents and paper-and-pencil.

#### 2.2.2. Quantitative measures: inclusion criteria and sample description

Multimorbidity was assessed by the count of the chronic conditions diagnosed. As outlined above, patients with two or more chronic conditions were categorized as multimorbid and eligible to join the study. The conditions were taken from the hospital electronic medical records as well as the number of drug intake of a patient per day. As a mental health indicator, the Patient Health Questionnaire-9 (PHQ-9) depression module was assessed in both partners. This is an established screening of depressive symptoms that is part of the *Patient Health Questionnaire* (Löwe et al., 2004). Values over 9 are seen as indicating subliminal levels of depression; the cut-off indicating clinically relevant depression is 15.

Subjective health status was assessed using a commonly used single item which also is part of the SF36 health survey (Bullinger et al., 1995). This item has been widely used in other studies as a proxy for subjective health (Cleary, 1997; Benyamini, 2011). The wording of the item is the following: “In general, would you say your health is…,” which is rated along a scale of “excellent,” “very good,” “good,” “fair,” and “poor.”

Functional status was measured using the *Self Care Index* at discharge; this is an assessment tool used as an indicator for the severity of nursing dependency (Koch et al., 2020). The Self Care Index is a score including 10 items from the 52 items of the “result-oriented nursing-assessment acute care” (ePA-AC, Hunstein, 2009; Koch et al., 2020) implemented at University Hospital Zurich. Index scoring (sum points) ranges from 10 points (fully dependency) to 40 points (full independency).

### 2.3. Qualitative analysis

Qualitative analyses followed the framework of grounded theory as suggested by Strauss and Corbin (1994). The data were analyzed using a modified grounded theory approach to identify categories of co-regulation to respond to the challenge of multimorbidity as in other mixed method studies (Höltge et al., 2018). In a first step, two authors of this paper (BMH, ABH) independently open-coded transcripts to develop an initial set of codes that were then grouped and embedded in existing theoretical frameworks.

In a second step, we changed between axial and selective coding by defining more codes, grouping them, and discussing and clarifying the categories that we assigned to them. In a third step, we compared the defined codes for consistency. Next, we discussed and consolidated them all for the final codebook. During this discussion, codes were grouped, and main categories and subcategories emerged that led us to the formulation of a conceptual model after an iterative process of discussing, editing, and consolidating the categories. Consensus was achieved by discussions. Our procedure reassembled a thematic analysis (Braun and Clarke, 2012) approach. As in the mixed-method project the number of interviews was fixed within the procedures there, theoretical coding and theoretical sampling was not part of the procedure. We followed other examples arguing that around 15 interviews commonly lead to saturation (Guest et al., 2006).

## 3. Results

Table 2 presents all coded interpersonal emotion regulation strategies with a characteristic example sentence. As the interviews were conducted in German, these are translations. A broader selection of examples of coded sentences can be found in the Supplementary material.

**Table 2:** Types of categories in the three strategies in the couples as identified in the interviews, explained with example sentences.

Name and number of the category	Example sentences	Partner ID	Main categories Processes
Sub categories Individual strategies of the partners			
2 Self-care	…and I have to keep telling myself that I’m going to take the liberty of going out into nature now… because for me that’s the elixir of life.	PAR 02 m	Behavioral
7 Letting go and shift attention away	You have to be able to forget it, just forget it for the moment, otherwise you think about it every day and that’s not good either.	PAT 01f	Cognitive
11 Reliance on other close relationships	Obviously, what I want to see are my grandchildren… they are anchors that you hold on to.	PAT 09 m	Behavioral
	…the reliable, good ones (friends), who know the situation and who have acted appropriately and have been helpful. And that is really a support for us, so that you know, yes, you are somehow safe.	PAT 04 m	Behavioral
15 Dealing with fear, sorrow	I can also cry sometimes and so on…then I have to say to myself:” Now you have had enough whining, now we will move on again.”	PAR 02 m	Cognitive
We-ness shared – overlapping strategies			
4 Shared positive focus/humor/ease	PAT: You drove me everywhere in the wheelchair (in hospital) – PAR: Yes, well I raced you in the corridors (they look at each other and laugh).	PAT/PAR 01f	Cognitive/Behavioral
	Always trying to see the positive…and every day is a new day and there is something that makes you happy. Walking together like this is beautiful too.	PAT 02f	Cognitive
6 Focus on the essential/meaningful, intensifying life	There are things in life that are no longer so important. You have to get rid of them and concentrate on the essentials, and then you gain a little time. I think it is also very important to have time for each other.	PAR 03 m	Cognitive
9 Trying to maintain normality	There should be still room for socializing, for a glass of wine and a good meal…I think that is important…that there is a certain normality, not just focusing on the illness.	PAR 02 m	Behavioral
10 Acceptance/go with the flow of things	this is….Yes, like a little bit familiar with catastrophes, with deterioration, with illness. So that you do not see it as an emergency but maybe as a rule, which comes every now and then. This is an important attitude as a couple.	PAR 04 m	Cognitive
12 Keeping the fighting spirit and cultivating strengths	I fight and we fight together because I want to stay in this world as long as possible.	PAT 10 m	Cognitive
13 Maintaining hope, spiritual references	With the certainty that “we are in God’s hands,” we can master many things better.	PAR 13 m	Cognitive
14 Factional approach	I think it helps that we can both be a bit more matter-of-fact about things, sort things out and not to fall into a subdued mood too quickly.	PAR 10f	Cognitive
Relationship related strategies			
1 Showing gratitude/appreciation mutually	He did everything for me, he helped me through many difficult times. In fact, you do not find a partnership like that anymore. I would certainly do the same for him, it is mutual.	PAT 01f	Interpersonal
	PAT: Yes, I’m proud of you, too… You’re doing very well. PAR: This is my job. PAT: Hm… not necessarily…I must say, my partner has incredible strength. I’m proud of that, too.	PAT and PAR 02f	Interpersonal
3 Dealing with the threat to equity in the relationship	Sometimes you reach the limit a little bit. Yes, it is not always that easy because you do not have a partner, you have a patient.	PAR 09 m	Interpersonal
	If it is going to be sustainable in the long term, then it has to be mutual…at some point, you feel exhausted or feel doubly burdened. So, it needs a give and take.	PAR10f	Interpersonal
5 Disclosure of thoughts and feelings, culture of honesty	The most important thing is to always say clearly when something is not good or when you should do something.	PAR 10f	Interpersonal
	…to be honest … to talk openly, to approach each other, of course.”	PAR 03 m	Interpersonal
8 Strengthening the common ground in the couple	The worse we feel, the more intensively we need to stick together.	PAT 10 m	Interpersonal
	…that you stand together. That you help each other, encourage each other.	PAR 05f	Interpersonal
16 Balance of autonomy in the couple	By giving each other freedom, we have noticed that, that is really very nice that he can use his own freedom and I can do the same and we also have things in common.	PAT 10f	Interpersonal
17 Compensation: The partner compensating own deficits	That’s when you need the driving force with the whip at the back… to get me back on my feet and say: do it now and do it now and do it now and otherwise….	PAT 09 m	Interpersonal

**Figure 1:**
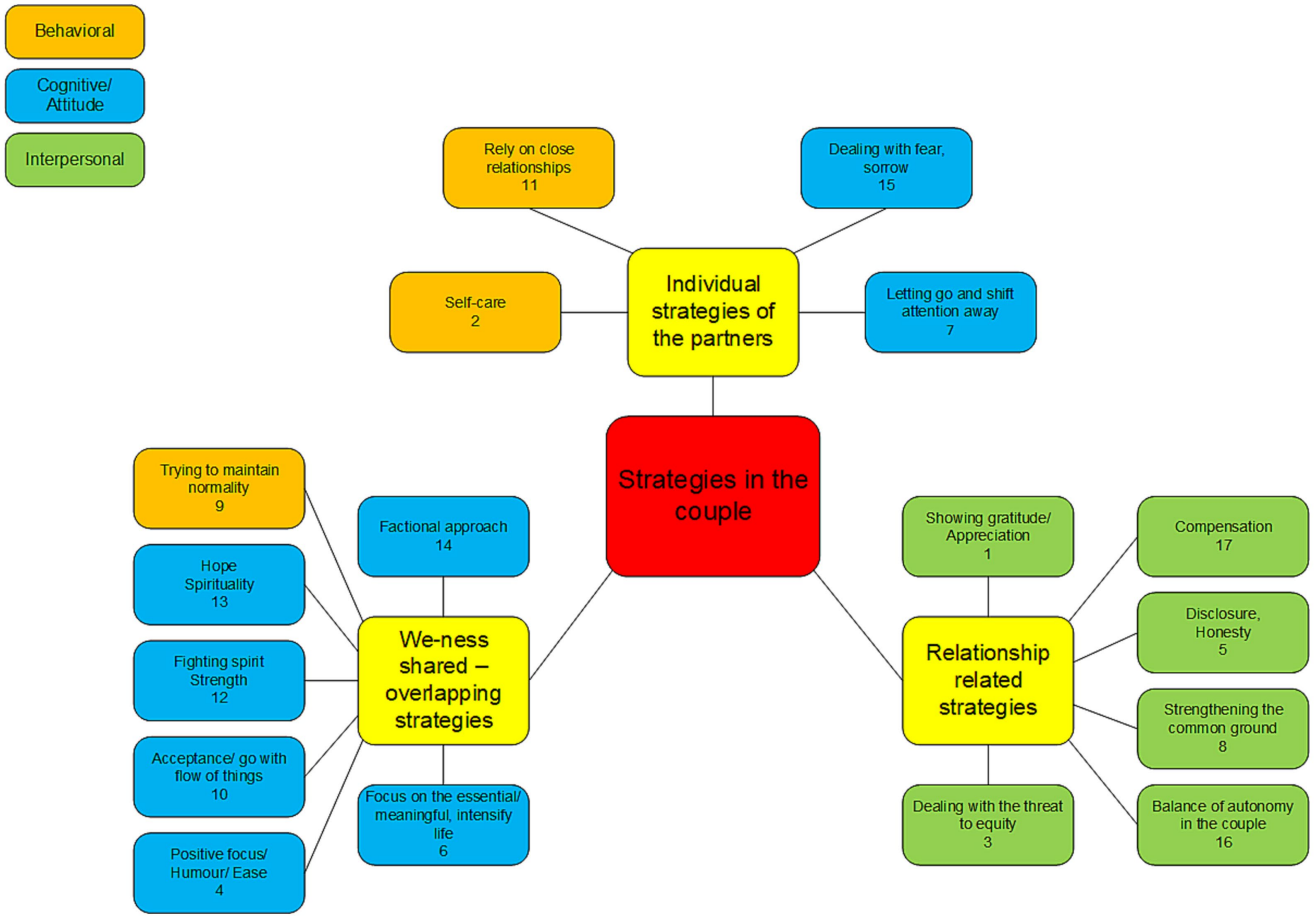
Conceptual framework, categorized by the defined main categories of strategies: We-ness and shared or overlapping strategies, relationship related strategies, and individual strategies of the partners. The numbers in the specific categories refer to the categories as depicted in Table 2 and emerged from the order of analysis.

Number: The numbers in the specific categories emerged from the order of analysis and correspond with the numbers in Figure 1. Partner ID: PAT, Patient; PAR, Partner [couple ID]; Main categories processes see conceptual figure (1). More coded sentences of the interviews are listed in the supplement.

During the interviews different perspectives on how to deal with the situation of an acutely hospitalized partner with multiple chronic diseases came up. We developed a conceptual figure from the emerging categories (see Figure 1). As an ordering higher-order structure, we allocated the categories according the psychological process involved in the coping strategy and color-coded them in the conceptual figure, as *behavioral* (orange), *cognitive* (blue), and *interpersonal* processes (green). First, *behavioral* strategies were coded when the theme mentioned concerned strategies that mostly involved behaviors and actions like meeting people, performing positive activities like going to a spa, or maintaining a regular daily schedule to maintain normality even in times of chronic crises. Second, when couples referred to their attitudes and *cognitive* appraisals toward the situation, categories were assigned to this category. These individual appraisals included categories often referred to in the literature, such as fighting spirit, positive or optimistic focus, meaning seeking, and acceptance. We included strategies that involved cognitive emotion regulation strategies (acceptance, positive reappraisal, etc.) as well as spiritual strategies (faith that God makes all things well) and humor in this category, given their mostly cognitive components. The humor category was based on a couple referring to a strong resource they based on sharing a humorous and playful atmosphere amidst disease and crisis—their example of a wheelchair race in the hospital hallways surely included behavioral components of playfulness. These categories were not genuinely relational but were mostly explicitly referred to as shared between the partners. Third, we coded strategies that were *interpersonal* in nature and referred to relationship quality and closeness as interpersonal (green colored in Figure 1). Some strategies might combine interpersonal, behavioral, and cognitive processes; when the process was genuinely interpersonal, we put the label interpersonal as most important feature for the research question.

Categories on these different process levels again could be ordered along three higher categories characterized by the *relational perspective* of the shared process of interpersonal emotion regulation in the couple. Overall, couples reported a positive and grateful view on how they co-regulated and coped together during the complicated health situation of a current crisis upon the background of multimorbidity. There was one exception: One female partner of a patient reported bitterness and a pronounced feeling of being misunderstood by the partner in how the situation overwhelmed her and demanded investment of effort, energy, and time. In this interview the interviewer was not able to structure the interview in a way that allowed both partners approximately equal speaking time. The following three sections present main categories and they corresponding categories.

### 3.1. We-ness related and shared strategies in the couple

First, *we-ness related* strategies were identified. The categories allocated here involved attitudes and actions that were reported as *shared within the couple*. The quality and the semantics of these appraisals differed; from underlining shared fighting spirit, to referring to maintaining humor, ease, and playfulness together as a way of coping with the situation, different categories emerged (see Table 2). They shared the characteristic that *both partners* referred to them and identified them as characterizing how they dealt with the complex health situation *as a couple*. These expressions were conceptually allocated in this we-ness category, as they share the characteristic of co-constructing the disease situation on a dyadic level, transmitting a shared view, with no individual differences in the couple evident. The interview was set up to explore these shared views as both partners were interviewed together. Couples were asked what helped them to deal emotionally with the situation, most couples could verbalize a shared ‘ideology’: There were references to a shared faith in a higher spiritual power that takes care, or the very factual perspective of adhering to a matter-of-fact thinking style in order to stay calm. Further, some couples explained that certain things became less important during the trajectory of the disease and that they now focused on meaningful things, essentials like sharing and enjoying time together (see Table 2 and example sentences “focus on essentials…”). We also included the behavioral strategy of maintaining normality in this category, as it was referred to as a shared focus in the couple. Maintaining one’s own projects and framing the maintenance of a sense of normality was seen as a resource and need. Fighting together for a longer lifetime together was one strategy mentioned, reflecting a fighting spirit and a ‘never give up’ attitude. In contrast, other couples spoke of a less active, accepting attitude, saying that things flowed, that catastrophes came and went, and that taking it as it was helped them the most in coping with the repeated occurrences of health complications in the multimorbid partner. In the couples that had a negative view of their coping process, no shared attitude was expressed.

### 3.2. Relationship-related strategies in the couple

During the interview, when asked how they dealt with the situation emotionally, many couples articulated relationship-related aspects. Relationship-related strategies share the characteristic that these strategies are aimed at improving relationship quality and closeness. In contrast to the shared and we-ness related strategies they are not based on dyad level shared, construal, but individual perspectives targeted on the relationship. Here the asymmetry of the “multimorbid patient vs. care-taking partner” situation is often referred to, and the experience of the situation include explicitly different perspectives on the situation and the strategies. As the interview was conducted with both partners together, talking about these aspects often represented disclosure of very emotional and touching content for the couples, thus giving space for the different perspectives of both partners.

Sincere gratitude was expressed several times by patients to the partner for always being there. Gratitude and appreciation for the relationship and the strength of the partner was referred to as mutual and as something precious. Moreover, the importance of cultivating and strengthening the common ground of the relationship by expressing mutual appreciation and ‘sticking together’ was considered important—a ‘give-and-take’ that was balanced and strong. Being honest and disclosing not only positive but also negative feelings was also articulated as an important foundation for co-regulating this demanding situation. Again, in contrast to this view, one unhappy female partner took space to utter complaints about not being seen and understood, while the partner felt like a burden and the marital frictions as a major source of distress.

Mirroring this, other categories expressed touched on the challenge of dealing with the asymmetry in the couple, given the roles of the patient and the partner, who is at least in crisis situations also a caregiver. Also touching on the give-and-take perspective, some patients experienced their partner as a driving force who was there to spur them on when their own strength and motivation dwindled, in the sense of a compensatory function of the partner; when one’s own strength dwindled, the partner stepped in. Others explicitly referred to the challenge of maintaining mutuality as well as the threat of equity, given the confusion of roles between being romantic partners to each other and at the same time being in a patient/caregiver relationship. Also mentioned was mutuality in the couple as a way of sustainability that was required given the chronicity of the situation. To find the necessary balance in the couple, many couples referred to the importance of giving space and freedom to the partner, so that each could independently activate resources and recharge their batteries. In line with this, one female caregiver explicitly complained of a lack of correspondence of effort and a lack of mutuality while most other couples acknowledged the challenge but seemed to be mastering it. Here the communicative situation seemed stuck, the asymmetry of give-and-take was tangible, and the caregiving wife was very repetitive in her way of complaining about the situation. The rigid, repetitive way of sharing her feelings about not being seen in her exhaustion reassembled characteristics of ruminative co-brooding (Horn and Maercker, 2016). Moreover, the partner did not seem to be perceived as responsive by his wife. As mentioned above, responsiveness is defined by the shared notion of being understood, validated, and cared for Reis and Shaver (1988) and tends to be limited in situations of disclosing emotional content with the repetitive, rigid quality that characterizes ruminative thoughts.

### 3.3. Individual strategies of the partners

Third, partners mentioned *individual strategies* that explicitly involved actions without reference to or presence of the partner, such as self-care, reaching out to close others, or dealing with their own worrying about the disease and their partner’s well-being by letting go and shifting attention away from the topic. These were strategies characterized by not including the partner and focusing explicitly on cultivating resources that lie outside the couple relationship. They included behavioral strategies involving relaxation and self-care activities as well as the activation of social relationships outside the romantic one, such as relationships with children and grandchildren as well as close friends. As we approached the couples when the partner with multimorbidity was hospitalized, some couples emphasized the importance of using the hospital stay as a chance to take time for self-care and cultivating other relationships. Further, the importance of individual cognitive processing and attention allocation when dealing with anxiety emerged as a theme. Fears that the partner may become disabled or die are difficult to share within the relationship, and there were references to shifting attention and letting go of one’s own negative emotions in the context of the complex health situations.

## 4. Discussion

The goal of this study was to explore emotional co-regulation of couples adjusting to a complex health situation characterized by multimorbidity. The couple’s statements revealed a picture that in many ways mirrors concepts and findings in the literature: The health situation always affects both partners in a couple, adaptive services must be provided by both partners, the concept of we-disease can be described as supported (Kayser et al., 2007). Co-constructing the situation in shared appraisals that shape the emergence and regulation of the emotional response as well as coping with the stress challenges proved to be central in our study as well, in line with the literature.

At the same time, the individuality of these appraisals is striking. This accords with appraisal theories in affective science explaining how one and the same situation can provoke such different emotional responses. Almost all couples interviewed put into words a common attitude that helped them find a way to deal with the situation; however, the content of these appraisals differ significantly. From a fighting spirit to an almost fatalistic trust in divine providence, trust that everything is in flux and that crises come and go, to an attitude of interpreting things very factually and dryly with playfulness and a sense of humor, the couples in our study report semantically different strategies. Interestingly, positive relational processes like sharing a playful, humorous attitude are in line with recent claims for the importance of positive resonance in close relationships as an important source for psychological well-being and flourishing (Frederickson, 2016). This is in so far conceptually important as it broadens the view from focusing only on the mastering of demands in the disease situation to cultivating positive emotions by relying on co-regulatory processes that drive resonance of positivity in the couple. In total, all couples more or less explicitly underline the importance of sharing the attitude and emphasize that this shared view of the situation and how to cope with it gives them strength and peace. Sometimes only the general importance of these aspects is mentioned; sometimes deep gratitude is expressed by the patient for actually receiving them.

Most of the couples point out that dealing with the unstable health situation of multiple chronic conditions, including acute health crises, has led them to a more conscious and closer life together. They often speak of enjoying time as a couple more consciously and focusing on meaningful aspects of life. Many patients and their spouses mention that time with their families and friends and feeling supported by them are further important strategies for dealing with this complex situation. This is in line with the seminary Socioemotional Selectivity Theory of aging by Carstensen et al. (1999), which postulates that when time is perceived as limited, meaningful social interactions are prioritized and less meaningful activities implying less rewarding outcome are neglected. Another theme emerged that might be seen as conflicting but perhaps also characterizes a common tension in extreme situations: In the turmoil of health-related challenges, couples point out the importance of trying to maintain normality in everyday life. Bridging the madness of the situation and the need to prioritize meaningful aspects of life with the need to keep up a normal life with mundane activities and mundane social exchange has been conceptualized as a core source of relational regulation underneath the benefits of social support (Lakey and Orehek, 2011). For the couples, this again is an area for synchronizing or negotiating a balance that works for both individuals in the couple.

Only three couples in our sample offered a negative picture of their shared regulation and coping process; interestingly, their reports displayed some contrast to the topics raised in the other interviews: They mentioned no shared attitude, no feeling of being seen and understood, no mention of a co-construction of the reappraisal of the situation, and no sense of growth or meaning in the situation. Furthermore, their utterances within the interview were repetitive and had a negative focus and thus showed a ruminative quality. The social transmission of ruminative blockades into the relationship associated with unsuccessful individual emotion regulation and worsened relationship quality was reassembled in this one couple, as the literature on co-rumination would suggest (Horn and Maercker, 2016). Further research is needed to gain more insights in the predictors of this dynamic, as it has very important implications for couple interventions in this area. Possibly, a co-ruminative dynamic within the couple could be seen as an indicator for starting with individual psychological support before strengthening the relationship quality is considered.

From a relationship research perspective, the overlap or sharing of attitude is what constitutes closeness in the relationship (Berscheid, 1994). Close relationships have been defined as being constituted by an interdependence of thoughts, attitudes, and actions that allows expansion of one’s own resources to include those of the partner (Aron et al., 2004). This expansion of resources, however, presupposes care and cultivation of the quality of the relationship (Manne and Badr, 2008). This is also mentioned by the couples, who emphasize openness and honesty and the work on the common ground in the relationship. As mentioned above, when coping with diseases the significance for better outcome of an overlap of appraisals like illness perceptions (Sterba et al., 2008) and dyadic coping (Meier et al., 2019) has been proven earlier in the literature, in line with our findings.

The cultivation of common ground is reflected in the relationship-related strategies that the couples spontaneously mentioned. They are in line with dyadic views on we-disease (Kayser et al., 2007; Leuchtmann and Bodenmann, 2017) and the conceptualizations of the establishment of psychological intimacy as a generic constituent of relationship quality (Reis and Shaver, 1988). In the latter, the importance of disclosure and responsiveness – being understood, cared for, and validated- as constituting the interactive process of maintaining intimacy has been underlined and has been applied to the context of coping together with illness and disease: Maintaining closeness and a mutual notion of being understood and validated is particularly demanding in an asymmetric situation when one partner has a disease. In line with social psychology views on equity or mutuality in the couple that has been applied to couples coping with different health conditions (Kuijer et al., 2004; Manne et al., 2004; Kayser and Acquati, 2019), some couples speak of the challenge of maintaining closeness in the asymmetric situation of one partner being affected directly by the disease and the other only indirectly. Interestingly enough, in their explicit framings of how to deal with multimorbidity in the couple, they emphasize the importance of giving each other space to activate individual resources outside the couple. This was possibly particularly triggered by the hospitalization situation, which gave them the freedom to see their own friends, play with their grandchildren, get outdoors and enjoy nature as ways of taking care of themselves. However, as most patients in the sample were rather independent and still functional in their daily activities, the idea of providing space for the partner to cultivate their own coping resources, social and individual, was not limited to the hospitalization situation. This is in line with the conceptual reasoning that has been introduced concerning the interplay of intra- and interpersonal regulation of emotions. A certain level of adaptiveness in the way each partner deals individually with their own emotions fosters the likeliness of successful co-regulation. Taking care of oneself as a way of taking care of each other in the couple and flourishing is intuitive and is mentioned by many couples. This is in line with findings and concepts in the literature (among others Willi, 1997; Horn et al., 2021).

It is important to note that following the enabling hypotheses of social support, there is also an effect to assume in the opposing direction: Functioning dyadic regulation also might enable individuals to improve their self-regulation, an association that recently has been found in cardiac patients with increasing self-efficacy as a mediator (Rapelli et al., 2022). In contrast, in another study, dyadic coping as a relational process mediated the effect of self-efficacy to marital quality in couples facing lymphoma (An et al., 2021). Integrating these and other findings, it is plausible to assume a bidirectionally working interplay of building and exploiting individual and dyadic coping resources.

Keeping up autonomy, not only of the patient but also of the spouse (e.g., by taking advantage of the hospitalization for doing ‘our own things’ or spending time apart with their children), was a further topic the couples often mention. Spouses highlight the benefits of keeping up their own activities or even seeking professional psychological support for themselves. This is interesting, as the health system often does not even offer psychosocial support to patients, much less to their partners. However, fostering adaptive emotional processing in the partner, even independently, might be a promising way foster resources for relational regulation, which in turn results in better adjustment in both partners. This might be the case particularly in older couples, as the relationship is characterized by even more profound interdependency, but at the same time, arousal vulnerability (Charles, 2010) and thus contagion of emotional responses might arise. This negative contagion effect of partner distress has recently been found even on the level of proinflammatory gene expression of the listening partner (Wilson et al., 2023). In other words, more closeness in couples in late adulthood is not only a resource, it concurrently challenges partners with an added regulation task at hand resulting from co-suffering with the patients.

Many patients highlight the value of their spouses’ affective support by providing opportunities for talking openly and honestly with each other, simply being there, being reliable, and fostering a positive reappraisal of the situation, which is line with earlier findings on disclosure by Manne and Zautra (1990). Some couples underline the value of being strong and fighting together; others focus more on shared humor and light-heartedness within the difficulties. The interview seemed to be a safe space to speak about topics that Swiss couples in the cohorts in the sample do not often explicitly discuss. This led to touching moments in the interviews of sincerely expressed gratitude and solidarity that involved the sharing of deeply personal and emotional content.

### 4.1. Co-regulating multimorbidity: for better, for worse…

The categories that emerge in our study illustrate how couples deal with this challenging situation and point to the importance of not only supporting maintenance of the partners’ autonomy but also of feeling responsible for self-care in order to cope with the additional strain of being the caregiver. This study followed a dyadic perspective, interviewing the couple together. This interview set-up surely provoked different outcomes as compared to individual interviews. It is possible that some aspects were not spoken about openly in order to protect the partner. Our study reflects the co-construction of the situation in the couples and might differ from the individual situation when investigated in individual interviews.

However, these shared appraisals have been identified as fundamental when it comes to taking advantage of the resources the evolve out of romantic relationships in times of disease (Berg and Upchurch, 2007). The concept of a protective co-construal of a we-disease as a shared yet manageable situation has been supported in many studies that found that the we-perspective on coping with the disease predicts better outcomes (Rohrbaugh et al., 2008). Further, the inclusion of the other in the self, the construal of couple-level identity, expands those resources for the individual not only by including the partner’s resources in the form of instrumental or emotional social support: The new systemic WE-entity is a resource in itself by providing connection and belonging. This not being alone when facing a stressor is a value in itself and has been identified as an emotion regulation resource. Social Baseline Theory suggests that not being alone is a cue for a more predictable and controllable environment and invites a person to save their own resources by relying on the other (Coan and Sbarra, 2015). This theory has been informed by functional magnetic resonance imaging (fMRI) studies revealing that top-down regulatory networks that involve effortful emotion regulation are downregulated during threat of electric shock if individuals are holding their partner’s hand (Coan et al., 2006). To sum up, a systemic perspective suggests that we-ness, the overlapping co-construction of a dyadic identity, represents more than the sum of the individuals and is reflected in the statements of couples.

This is true for better and worse: Not all couples interviewed expressed gratefulness and a positive view on their dyadic processes. For some, relational frictions resulting from being overwhelmed by the situation were not resulting in the perception of support and resource activation; in contrast there were seen as an additional burden beside the complex health situation. An example of utterances in the interview of one couple illustrates this impressively: One couple in which the female partner expressed unhappiness with the situation made explicit reference to the lack of own space and resource activation. In contrast, they mentioned the burden of too much to deal with as not leaving space to breathe. The female partner framed it this way: “At the moment I’m really at the limit… I really have to find a way to be more selfish…and just, it (caretaking for relatives) has no value in society…If you run a marathon or something… yeah, that’s great what you did. But if you take care of seriously ill people at home, then… yeah, nobody wants to hear about that because it’s not interesting.” It is worthwhile to note, that here not only the appreciation of the investment and the lack of equity by the spouse is a topic, but rather the lack of societal acknowledgement, a factor that has been discussed in the stress-response literature to be important for successful adjustment to major stressors and trauma (Maercker and Horn, 2013). The spouse (suffering among other diagnoses from Chronic obstructive pulmonary disease (COPD), a progressive lung disease associated with increasing limitation of lung ventilation (airflow)) in turn uttered in the interview “For me, my mood depends mainly on two things… my acute condition in relation to air and that is sometimes not so easy. The second thing is arguments with my wife, which gets me all worked up. That weighs on me…I cannot breathe then either and …that’s almost worse than like an infection. That’s why I keep quiet most of the time… if one gets the feeling that it would probably be better to leave now. Because for everyone else a burden would be gone.” The partner immediately replied “: I do not think we give you that impression.” These utterances from the interview illustrate how co-regulation in dyads can overwhelm the couple: A feeling of being misunderstood, not being seen (by the partner and society), the spilling over of individual unsuccessful emotion regulation into the co-regulation, exhaustion mixed with a very negative view on oneself, the social context, and the future (nicely reassembling Beck’s depressive triad; Beck, 1979), a double lose situation for individual and dyadic regulation of emotional responses, being alone together. When couples find themselves in a place like this, offering support needs to adjusted. There are voices in couple research claiming a taxonomy of couple functioning; that means that relationship quality might not be a merely dimensional construct but taxonic between functioning couples and couples in discord (Whisman et al., 2008). This has significant clinical implications: It is to be expected that couples in discord facing multimorbidity or other diseases are need of a different quality of psychosocial support offers than non-discordant couples. Another indication of different individual needs spilling over into the couple’s functioning might be elevated depressive symptoms that easily can be screened. In these situations, starting by providing individual support to the partner is possibly warranted to address different needs. This is in line with the idea that a certain level of individual emotion regulation success relying on one’s own resources is required to foster beneficial relational processes. A lack of equity can be integrated in the self-image and couple image, as our couples refer to explicitly in their interviews. However, lack of space for individual development while being seen by the partner might be a situation that is associated with less well-being in both partners and a less beneficial outcome. In couple therapy, a successful balance between individual growth and staying connected in the relationship has been referred to as “related individuation” (Willi, 1997). There is consensus that the quality of the relationship is an important predictor of outcome, as other studies examining relationship quality and health outcomes (Kayser and Acquati, 2019; Stanton et al., 2019) suggest. For chronic patients who often need care, not only the balance of equity is threatened; they often feel like a burden, with all the involved implications for their individual well-being und relational functioning (McPherson et al., 2007). Accordingly, there is a call for interventions that foster the necessary components to cope with disease and improve well-being in couples facing diseases (Baik and Adams, 2011; Rohrbaugh, 2021).

### 4.2. Limitations and particularities of the sample

During the recruitment period we approached *N* = 515 couples who did not consent to participate in the study. As is common in other couple research, the sample seems to represent a selection of rather well functioning, happy couples—with the exceptions mentioned above. The ratio of functioning and non-functioning couples in this sample cannot be interpreted as representative for all couples facing multimorbidity, nor can the results of the qualitative analysis. More representative sampling of different couples, not only in terms of relationship quality but also other groups with different cultural and socioeconomic backgrounds and living conditions as well as not heteronormatively identifying couples, is needed in order to obtain a better understanding of the generalizability of research findings in this field and their clinical implications. Furthermore, we did not follow a classical grounded theory informed strategy of concurrently performing theoretical coding and sampling. The saturation of our data referred to the literature and coincided with the overlapping content that informed the categories we suggest for further conceptual development. Our procedures overlapped with thematic analysis an approach the aims at extracting themes from a given sample of texts without the deep-thinking philosophical assumptions and recursive process of theoretical coding and theoretical sampling that grounded theory has (Braun and Clarke, 2012). Therefore, our categories could be framed as themes. This is an important point and we hope that our strategy nevertheless resulted in heuristically valid insight in the experience co-construction of dyadic adjustment to multimorbid situations of the couples in our sample. Our aim was to embed the documented experiences in existing theories and findings in the field and thus a theoretical elaboration. We state this inductive and deductive transparently as contrasting to grounded theory philosophical underpinning also acknowledging current discussions of the epistemological and methodological challenges of qualitative research in health psychology (Braun and Clarke, 2023).

Medical diagnoses differed in this sample, and we did not focus on the different medical regimes that needed to be followed, nor on the different ranges of required health behavior changes. For more specific insight into the dyadic processes involving specific health behavior changes, a more consistent sample would be useful. In this study, however, we focused on the emotional processing and co-regulation of a chronic complex health situation that all couples shared, given the multimorbid situation. All couples shared the chronic time line and the complexity of more than one chronic condition and associated treatment regimes. Further, palliative patients were excluded, meaning that the conditions were not characterized by being fatal or implying an acute risk of dying. Some heterogeneity was also given in terms of the caregiver burden of the partner. It is important to note that most patients did not need caregiving in a medical sense by their partners, as the caregiving index suggested high to complete levels of independence. In terms of mental health indicators, one third of the patients but only one of the participating spouses reported an elevated level of depressive symptoms. As depression can be framed as dysregulated emotional responses, these elevated scores further underline how emotionally challenging the situation for some participants was.

It is important to note that our interview explicitly addressed the way couples deal together with the *emotional* impact of a complex health situation. The focus was clearly on emotional co-regulation and not on the commonly addressed topics such as caregiver burden (Revenson et al., 2016), dyadic coping (Bodenmann, 1997; Revenson and Delongis, 2011), or social support (Revenson, 1994). These phenomena hugely overlap but are not identical. That the caregiving index revealed that most participants did not require a lot of care in the medical sense is reflected in the interviews, which addressed equity and autonomy in the couple but not so much instrumental support or dependencies – with exceptions as outline above. As both partners were present, possibly caregiving partners hesitated to express their caregiver burden to protect and not upset their partners with the multimorbid situation. That might be seen as a limitation. At the same time, it may also be a strength of this study that the systemic perspective was consistently implemented and that the focus was on dealing with the emotional impact of the diseases. We found that even if the patients with the chronic diseases did not need any pronounced instrumental support from the partner, partners were just as emotionally affected by the situation as the patients, and some even more so. The situations did not contain a direct threat to life but still evoked anxiety and worries and a significant amount of psychological adjustment to a situation that is chronic and unpredictable. From an affective science perspective, it is plausible that this unpredictability and the chronicity of the situation evokes appraisals that lead to emotional responses. Coming back to the definition of emotion introduced above, emotional responses are there to react in an adaptive way to changing demands of the environment. A sense of lack of control and unpredictability evokes emotional responses; that is reflected in the statements by the interviewed couples and the elevated depressive symptom levels of some patients. This has relevant clinical implications in so far as often the focus is on the burden of instrumental caregiving and palliative situations. Without a doubt, these situations concern the couple as a system. Our interviews show that complex chronic diseases affect not only the patient but also the romantic partner, even if the stress burden of caregiving might be considered by medical standards as not yet very pronounced.

### 4.3. Conclusion

The present study supports a dyadic view on coping with multimorbidity in couples. Couple interviews revealed facets of co-regulation including disclosure, responsiveness, shared mindfulness, humor, co-reappraisal, and social proximity—strategies that are associated with interpersonal emotion regulation in the literature. The interviews also reflected a pronounced interdependency between the patient’s quality of life and that of their partner as well as a shared desire for autonomy. The interplay between intrapersonal strategies of the patients and their spouses requiring necessary space for activating individual resources and adaptive emotion regulation and the potential of successful co-regulation deserves further investigation. A better understanding of this logic seems to be crucial for developing successful interventions supporting couples facing multimorbidity.

## Data availability statement

The raw data supporting the conclusions of this article will be made available by the authors, without undue reservation.

## Ethics statement

The studies involving humans were approved by Kantonale Ethikkommission Zürich (Cantonal Ethics Committee Zurich, Switzerland). The studies were conducted in accordance with the local legislation and institutional requirements. The participants provided their written informed consent to participate in this study.

## Author contributions

AH, LZ, and BH conceived and designed the study. AH supervised the data selection, entry, and drafted the manuscript. BH organized the database. BH and AH performed the qualitative analyses. All authors contributed to the writing of the manuscript and gave final approval of the version submitted.

## Conflict of interest

The authors declare that the research was conducted in the absence of any commercial or financial relationships that could be construed as a potential conflict of interest.

## Publisher’s note

All claims expressed in this article are solely those of the authors and do not necessarily represent those of their affiliated organizations, or those of the publisher, the editors and the reviewers. Any product that may be evaluated in this article, or claim that may be made by its manufacturer, is not guaranteed or endorsed by the publisher.
