# Supplementary material for: The worse we feel, the more intensively we need to stick together: a qualitative study of couples’ emotional co-regulation of the challenge of multimorbidity
Source: Front Psychol. 2023 Aug 11;14:1213927. doi: 10.3389/fpsyg.2023.1213927 (PMC10450955; doi:10.3389/fpsyg.2023.1213927)
Supplement: Supplementary file 1 [file Table_1.pdf]

Supplementary Material

The worse we feel, the more intensively we need to stick together: A qualitative study of couples' emotional co-regulation of the challenge of multimorbidity

Andrea B. Horn, Lukas Zimmerli, Andreas Maercker, & Barbara M. Holzer

*** Correspondence:**
Corresponding Author Andrea B. Horn
a.horn@psychologie.uzh.ch

1 Supplementary Tables

Supplementary Table 1.

Interview guide (English translation)

**Introduction to the interviewer**
The interview should be conducted on the topic of joint coping behavior and interpersonal emotion regulation in couples in complex health situations.
It should take a timeframe between 5 and 15 minutes.
**Aim:**<br>**Both partners talk about their experience of dealing with their emotional responses together.**
If one partner does all the talking, ask the other partner for his or her point of view. E.G.<br>How did you experience this?
What do you think makes you strong to go through this together?
What do you think is most helpful when confronted with such a situation?
The conversation situation should be as authentic as possible.

Since the language use is to be analyzed, influence the way the couples talk as little as possible (especially the use of pronouns WE/YOU – I/ME, HE/SHE should just pop up). Therefore, always start with very open questions and only ask if necessary because the conversation is not getting going.
Video recording should be installed early for habituation. It is reminding the video taping before starting the questionnaires and then turn it on when the completion of the questionnaire is about to end.
The content of the questionnaire will be:<br>- Shared coping (resource oriented)<br>- Interpersonal emotion regulation

**Interview**
**Introduction to the couple**<br>Thank you in advance for taking part in our study. As you already know, the purpose of this study is to examine the role of emotional co-regulation and the role of the partner in dealing with complex situations related to illness. For this purpose, I will now briefly ask you about your individual way of dealing with your complex health situation.
One more comment: For the evaluation of the video it would be a great help if we could do the interview in High German. Would that be all right with you?
We are interested in how you deal with this particular health situation in the couple and what you do to get through it well together.
I would like to ask you again directly and hear in your own words how you experience it.
**(Question 1):** You are currently in a difficult situation.<br>**How did you find your way to deal with the situation together?**
**(Question 2): If you were to recommend something to other couples: What is the trick, what is important to get through and master such a situation together as well as possible?**
**Explanations:**<br>[If no one starts: address directly; can also be repeated in the later questions].<br>May I ask you as a person affected by the health problems: What helped you in particular? What would you recommend to other couples?

How do you see it as a spouse? Are the same things important to you, or what do you think was central for you?
**(Question 3): When you think about how you have coped with everything, what have you found particularly positive? What are you perhaps even proud of when you think about how you have coped?**
**Explanations:**<br>A situation like this can have a negative effect on your mood, and it can be quite emotionally stressful, both when you are ill and when you see your partner like this. Most people in this situation also feel anxious or a little nervous, sad or depressed, or worry a lot.
**(Question 4): What was it like for you? How did you manage to deal with this as a couple?**
What helped you to keep up the best you could?<br>What did you do to make it easier for your spouse or partner?<br>Can you perhaps give an example of a situation in which you dealt particularly well together, e.g. with fear or nervousness or sadness?

Supplementary Table 2.

Example sentences – listed according the types of sub-categories in the three strategies (main categories) in the couples

Number: The numbers in the specific sub-categories emerged from the order of analysis and correspond with the numbers in Figure 1. Partner ID: PAT=Patient, PAR=Partner [couple ID]; Higher order themes processes see conceptual figure (1). The sentences are translation from German language.

**Sub-category 1 Showing gratitude/appreciation**	**Group**
PAT 01f<br>He did everything for me, he helped me through many difficult times. In fact, you don't find a partnership like that anymore. I would certainly do the same for him, it is mutual.	Interpersonal
PAT 02f<br>PAT: Yes, I'm proud of you, too...... You're doing very well. PAR: This is my job.<br>PAT: Hm...not necessarily...I must say, my partner has incredible strength. I'm proud of that too.	Interpersonal

PAT10f<br>I was proud of him for discussing it (her illness) additionally with the doctor and for standing up for me, that made me feel proud.	Interpersonal
**Sub-category 2 Selfcare**	
PAR 02m<br>…and I have to keep telling myself that I'm going to take the liberty of going out into nature now... because for me that's the elixir of life.	Behavioral
PAR 01m<br>I went to the psychiatrist - to get psychological support.	Behavioral
PAT 02f<br>Because that is not a problem (when he is pursuing his hobbies), I have not lost my independence. I can also arrange myself well.	Behavioral
**Category 3 Dealing with the threat to equity in the relationship**	
PAR09m<br>Sometimes you reach the limit a little bit. Yes, it is not always that easy because you do not really have a partner, you have a patient.	Cognitive
PAT02f<br>...that your job and my illness - that then perhaps my concerns that I have, that all this is also too much for you. That's why I think it's important that you also have a balance. Because one day I will no longer be there and your life will go on.	Cognitive/Interpersonal
PAT02m<br>PAT: For me, my mood depends mainly on two things... my acute condition in relation to air and that is sometimes not so easy. The second thing is arguments with my wife, which gets me all worked up. That weighs on me...I can't breathe then either and ...that's almost worse than like an infection. That's why I keep quiet most of the time... if one gets the feeling that it would probably be better to leave now. Because for everyone else a burden would be gone.<br>PAR: I don't think we give you that impression either.	Interpersonal
PAR02m<br>At the moment I'm really at the limit…I really have to find a way to be more selfish...and just, it (caring for relatives) has no value in society... If you run a marathon or something... yeah, that's great what you did. But if you care for seriously ill people at home, then... yeah, nobody wants to hear about that because it's not interesting.	Interpersonal

PAR12m<br>...it's incredibly important to me (that the patient has taken over some of the household chores) because otherwise I'll have even less time, and it won't work.	Interpersonal
PAR13m<br>I'm actually not that proud, not at all... because I've sacrificed myself so much... and now he continues to smoke... and I'm not proud of it… then just have the thought that it was all pearls cast before swine…I would have done better off staying at home and looking for myself... there is actually nothing to be proud of...rather negative, isn't it?	Interpersonal
**Sub-Sub-category 4 Shared positive focus/humor/ease**	
PAT01f<br>PAT: You drove me everywhere in the wheelchair (in hospital) – PAR: Yes, well, I raced you in the corridors (they look at each other and laugh).	Cognitive/Interpersonal
PAT 02f<br>Always trying to see the positive…and every day is a new day and there is something that makes you happy. And it is nice to walk together like that…	Cognitive
PAT03m<br>And I think there is always a good dose of humor, that you don't take it too seriously, but that you see the positive side of things.<br>.	Cognitive
PAR06m<br>Then we just take things as they come... to make the best out of them and that will always be a little bit better. …at the end of the day it's the good moments that count for me.	Cognitive
**Sub-category 5 Disclosure of thoughts and feelings, culture of honesty**	
PAR 10f<br>The most important thing is to always say clearly when something is not good or when you should do something.	Interpersonal
PAR 03m<br>..to be honest ... to talk openly, to approach each other, of course.	Interpersonal

PAR 01f<br>Calming down and talking about it - close together.	Interpersonal
**Sub-category 6 Focus on the essential/meaningful, intensifying life**	
PAR03m<br>There are things in life that are no longer so important. You have to get rid of them and concentrate on the essentials, and then you gain a little time. I think it is also very important to have time for each other.	Cognitive/Interpersonal
PAT 02f<br>We try to live more intensively.	Interpersonal
PAR 09m<br>You have to try to do something during the days or the time when things are going reasonably well.	Cognitive
PAT07f<br>Carpe diem, I think that's actually almost the most important thing	Cognitive
**Sub-category 7 Letting go and shift attention away**	
PAT01f<br>You have to be able to forget it, just forget it for the moment, otherwise you think about it every day and that's not good either.	Cognitive
PAR04m<br>I am afraid of falling...I have my worries too...but I have to push it aside a little bit. here is no other way.	Cognitive
PAR05m<br>I also like to distract myself (volleyball, hiking, etc.) or play cards with my friends or just do something else where I do not always have to think about it.	Cognitive
PAT05f<br>...or while shopping or visiting someone or so on and then it just gets better again. You have to be able to let go.	Cognitive
**Sub-category 8 Strengthening the common ground in the couple**	
PAT10m<br>The worse we feel, the more intensively we need to stick together.	Interpersonal

PAR05f<br>That you stand together. That you help each other, encourage each other.	Interpersonal
PAT04m<br>I have the feeling that for our relationship itself this was even more strengthening than the relationship already is.	Interpersonal
PAR05m<br>I think, that you have a more intimate relationship, a closer one. You might feel that you belong together.	Interpersonal
**Sub-category 9 Trying to maintain normality**	
PAT02m<br>There should still be room for socializing, for a glass of wine and a good meal...I think that is important...that there is a certain normality, not just focusing on the illness.	Behavioral
PAR04m<br>.. that I still had my own projects and ... that I also tried to convey normality in this way.	Behavioral
PAR09m<br>I have a job that I like very much, I enjoy my work and I'm sure that helps too.	Behavioral
**Sub-category 10 Acceptance/go with flow of things**	
PAR04m<br>this is…. Yes, like a little bit familiar with catastrophes, with deterioration, with illness. So that you don't see it as an emergency but maybe as a rule, which comes every now and then. This is an important attitude as a couple.	Cognitive
PAR03m<br>The path is given to us, we have to take it and we try to take as positively as possible.<br>And we have actually had good experience with this so far.	Cognitive
PAT03m<br>And actually, I don't think about the illness too much. I let it come to me.	Cognitive
**Sub-category 11 Reliance on close relationships**	
PAT09m	

In fact, what I want to see are my grandchildren... these are anchors that you hold on to.	Cognitive
PAT 04m<br>…the reliable, good ones (friends), they know the situation and who have acted appropriately and have been helpful. And that is really a support for us, so that you know, yes, you are somehow safe.	Behavioral
PAR02m<br>I am lucky to have really great children who always support me and are always there and yes, we have it really good together.	Behavioral
PAT 09m<br>For me, there is only my family. That is actually the only thing that motivates me. Because …without my family I probably would have said (to myself): "Forget it".	Cognitive
**Sub-category 12 Keeping the fighting spirit and cultivating strengths**	
PAT10m<br>I fight and we fight together because I want to stay in this world as long as possible.	Cognitive
PAR01m<br>And I always said (to him): fight, fight, be a fighter!	Cognitive
PAT02f<br>I always find a reason to keep on fighting.	Cognitive
PAT10m<br>Without my Rita, I would have given up long ago. There were enough opportunities to give up on me.	Cognitive/Interpersonal
**Sub-category 13 Maintaining hope, spiritual references**	
PAR13m<br>With the certainty that "we are in God's hands", we can master many things better.	Cognitive
PAR05m<br>..that we have pulled ourselves together over and over again during this long time, and that we have always found hope again.	Cognitive

PAT04m<br>Well, my wife has always brought out the positive in me, even in moments when I could not believe it, and in this way has given me glimmers of hope. I was helpless.	Cognitive
PAT02f<br>But you get your strength from somewhere: from your partners, from your sons and from your faith.	Cognitive
**Sub-category 14 Factional approach**	
PAR10f<br>I think it helps that we can both be a bit more matter-of-fact about things, sort things out and not to fall into a subdued mood too quickly.	Cognitive
PAR07f<br>…is threatening ... we are both too rational ... we have also taken other precautions (patient directive, last will and testament), …so that we are not exactly swept off our feet in any case.	Behavioral/ Cognitive
PAR10f<br>...let's see what works: what does the doctor say, do we have to change something in the household, do we need a different mattress, etc.? …really trying to focus on what the next steps could be and not always saying "Ah, what did I do to deserve this, now I feel bad again".	Cognitive
**Sub-category 15 Dealing with fear, sorrow**	
PAR02m<br>I can also cry sometimes and so on...then I have to say to myself, "Now you have had enough whining, now we will move on again."	Cognitive
PAR03m<br>Of course, I have fears sometimes, that's clear... I don't know how to overcome these fears and then function normally in everyday life. It works very well.	Cognitive
PAT05f<br>I am not as relaxed as I used to be. And I always have to think, "Why are you suffering, or why are you suffering now? Other people are still fit and you are not".	Cognitive
PAT04m	

...my wife, from a very reasonable, caring point of view, often has to slow me down …and this fear, I feel it, of course, not entirely unjustifiably... she often has to slow me down, which depresses me that I am actually the reason for her fears, so justified fears.	Cognitive/Interpersonal
**Sub-category 16 Balance of autonomy in the couple**	
PAT10f<br>By giving each other freedom, we have often noticed that, that is really very nice that he can use his own freedom and I can do the same and we also have things in common.	Interpersonal
PAR05m<br>If I needed a month's vacation or something like that, you would also accept or understand that.	Interpersonal
Pat05m<br>...my wife is much more psychologically stable and stronger than I am, but there are still situations where I see that I am being asked. That is an interdependence.	Interpersonal

**Sub-category 17 Compensation: The partner compensating own deficits**	
PAT09m<br>That's when you need the driving force with the whip at the back... to get me back on my feet and say: do it now and do it now and do it now and otherwise....	Interpersonal
PAR 05f<br>And I think now that you know that I don't take it so easily either. I've always given you a bit of encouragement and shown myself to be stronger than I was, and now I think that you know that I'm actually not unstressable either, you're rather stronger than you were before.	Interpersonal
